# Oral Rehabilitation in a Patient with Major Maxillofacial Trauma: A Case Management

**DOI:** 10.1155/2012/267143

**Published:** 2012-07-09

**Authors:** Elif Bahar Tuna, Mehmet Ozgen, Abdulkadir Burak Cankaya, Cenk Sen, Koray Gencay

**Affiliations:** ^1^Department of Pedodontics, Faculty of Dentistry, Istanbul University, Capa, 34093 Istanbul, Turkey; ^2^Denta-Kid Dental Center, Bagdat Caddesi, Buyukhanli Konutlari, Suadiye, 34740 Istanbul, Turkey; ^3^Department of Oral and Maxillofacial Surgery, Faculty of Dentistry, Istanbul University, Capa, 34093 Istanbul, Turkey; ^4^Department of Plastic Reconstructive and Aesthetic Surgery, Emsey Hospital, Pendik, 34912 Istanbul, Turkey

## Abstract

Traumatic injuries may cause anatomic deficiencies in soft and hard tissues. These defects often result in the loss of attached mucosa and alveolar processes, which might reduce potential prosthesis support and require bone and skin grafting. As a result of major maxillofacial trauma, complete or partial avulsion of the palate may require extensive surgical and prosthodontic rehabilitation. The appropriate treatment for the maxillary defect demands a multidisciplinary approach by a team which consists of various fields of dentistry and medicine. The planning prostheses should replace not only missing teeth but also lost soft tissues and bone, and they should include the hard palate, residual alveolar ridges, and, in some instances, the soft palate. This paper describes the treatment procedures including plastic surgery operation procedures and prosthetic rehabilitation in a 19-year-old woman after her severe bicycle accident.

## 1. Introduction

Dental injuries in association with facial fractures are common in maxillofacial emergencies [1, 2]. The patient with maxillofacial defects resulting from motor vehicle accidents may have numerous soft- and hard-tissue injuries ranging from neurologic involvement to fractures and/or avulsions of the temporomandibular joint, maxilla, mandible, teeth, and supporting structures [1]. Skeletal fractures often are associated with the fracture of bones adjacent to the maxilla, as well as varying degrees of involvement of the overlying soft tissues such as the eyes, nasal airways, paranasal sinuses, and tongue [3].

Facial fractures are usually treated by reduction and immobilization or fixation of the fractured segments, followed by occlusal adjustments and restoration of missing teeth and soft tissues where necessary [4]. However, patients with large avulsion of the palate are rare, and the treatment requires a multidisciplinary and different approach with extensive surgical and prosthodontic rehabilitation. Lack of an anterior palate may result in esthetic and speech difficulties in the patient. The tongue is unable to make contact with a solid surface during these functions, and patients exhibit hypernasal, often unintelligible, speech [3]. Besides, the anterior palate avulsion causes swallowing, biting, and drinking to be extremely difficult.

There are several treatment options available for rehabilitation in cases of partial loss of maxilla including removable partial dentures, fixed partial dentures, crown and bridges, and teeth-implant supported prostheses [5]. The prosthesis should replace all missing oral structures including both hard and soft tissue in the traumatic area [3].

This clinical report describes the prosthetic rehabilitation of a patient with bilateral traumatic avulsion of the anterior maxilla treated with fixed zirconia prosthesis attached with gingival-colored porcelain. Modifications of the basic prosthodontic principles have been utilized along with conventional treatment methods and treatment is completed by depending on the patient's needs.

## 2. Case Report

A nineteen-year-old female patient who had severe facial trauma was referred for dental rehabilitation after a series of esthetic surgery operations. The patient's history revealed a blow to her face after falling off a cliff during mountain biking. Her initial evaluation in Emergency Service reported that her general condition was poor, and her hemoglobin value was 6 mg/dL with severe maxillofacial trauma and bleeding. The patient had an emergency consultation at the Department of Plastic and Reconstructive Surgery after a rapid hemodynamic stabilization and CT scans. According to the medical records obtained from her physician, she had a severe soft tissue injury and accompanying comminuted bone fractures on bilateral maxilla, zygoma, periorbital area, mandible, and nasal bones. Bone fragments were fixed with titanium plates and screws without bone grafting. There was also a posterior vertical split fracture on the hard palate extending anteriorly to both sides creating a mobile free bone fragment on the anterior maxilla. Those fractures were also fixed after reconstruction and then soft tissue repair was done. Complications were not seen in the early postoperative period; however, followup of the patient indicated bone necrosis on the anterior maxilla including the alveolar process extending to the palate. After debridement of the necrosis process, the defect was reconstructed with mucosal flaps and bony reconstruction was postponed. The patient refused the bone graft surgery planned for the repair of the defect on the anterior maxilla and had been consulted for prosthetic treatment.

**Figure 1:**
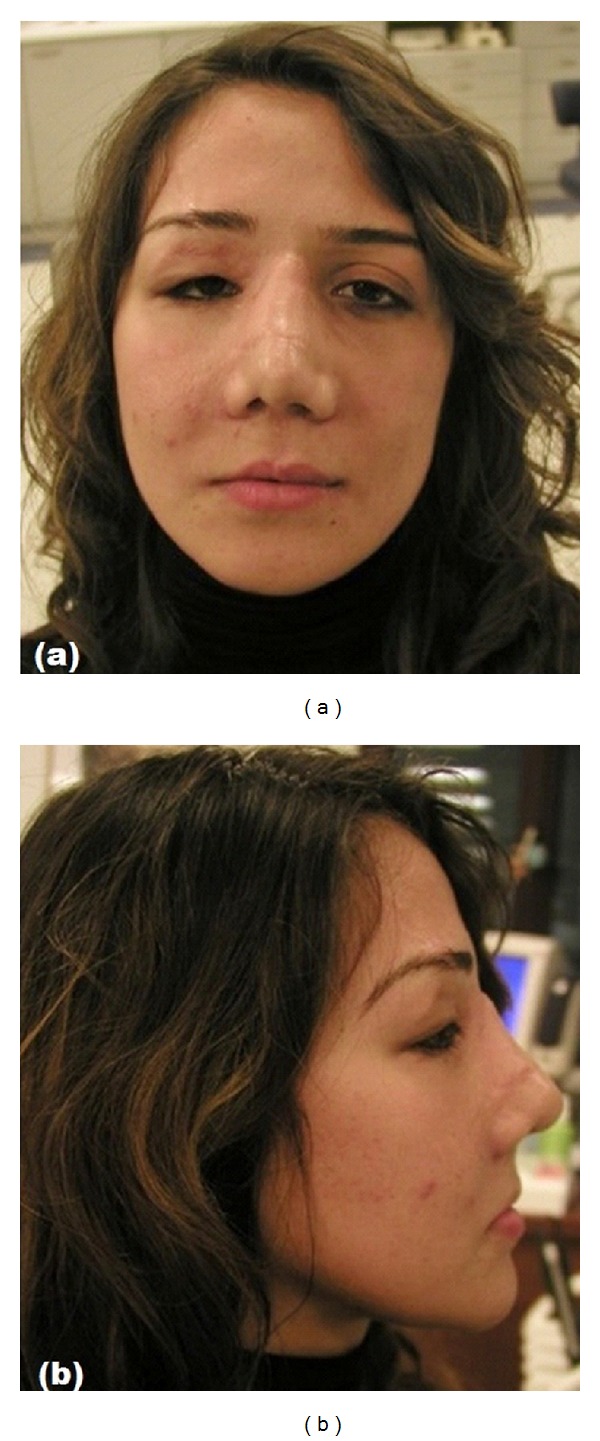
Facial view of patient before treatment with soft tissue defects on the eye area.

**Figure 2:**
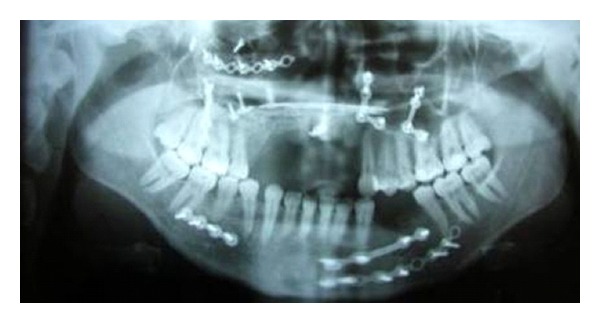
Panoramic radiograph showing mini plates and screws used for fixing fractured zygomatic arch, orbital, and maxillary sinus walls.

Her clinical examination showed soft tissue defects on the face particularly eye area and dysmorphic appearance (Figure 1). The panoramic radiograph demonstrated mini plates and screws used for fixing fractured zygomatic arch, orbital, and maxillary sinus walls. Intraoral examination revealed the absence of the anterior maxillary alveolar ridge and bone until the apex line; both maxillary central and lateral incisors and right canine teeth were lost as a result of traumatic injury (Figure 2).

**Figure 3:**
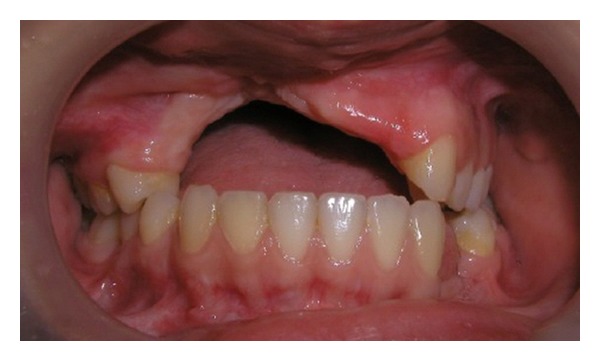
Intraoral view of patient before treatment, with missing maxillary and mandibular teeth and alveolar anterior palate.

**Figure 4:**
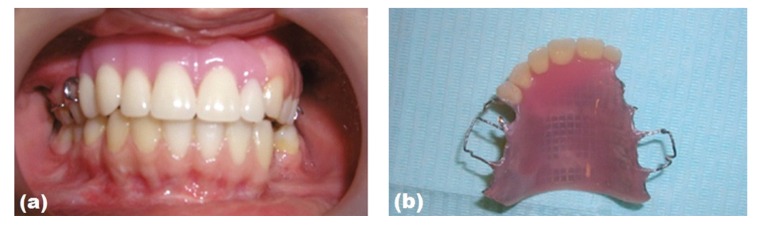
Intraoral view of temporary prosthesis with patient.

The patient had an Angle Class I occlusion with an acceptable vertical and horizontal overlap prior to the accident. Because of the loss of premaxillary segment, the patient experienced speech problems and had difficulty in biting and swallowing (Figure 3). In addition, the maxillary lip had lost support and was depressed into the defect area. The mandible was overclosed, resulting in a decrease of the vertical facial height. The temporomandibular joints were asymptomatic and jaw movement was in normal limits. The patient has complained of her inability to communicate, emotional disturbance of her appearance, and anxiety about the restoration of her teeth. After her extensive surgical procedures, initially temporary acrylic prosthetic rehabilitation was applied approximately one year later after trauma in order to restore her oral and dental function (Figure 4).

**Figure 5:**
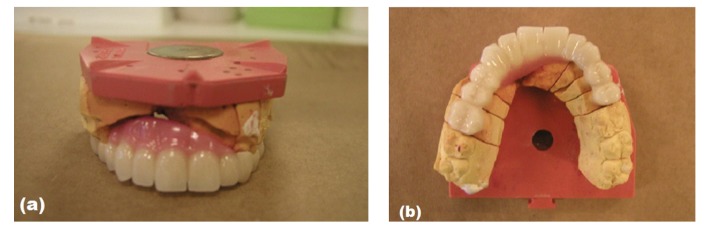
Framework of zirconia-based prosthesis modified with gingival colored porcelain.

As a treatment method, the zirconia-based crown bridge prosthesis had been planned and applied between right first molar teeth through left second premolar teeth for the replacement of the missing teeth (Figure 5). A new centric relation was made to transfer the articulator and shade was selected. This prosthesis was combined with gingiva-colored porcelain (Noritake Super Porcelain; Noritake, Nagoya, Japan) to compensate for the loss of hard and soft tissue on the anterior maxillary area and lip support. The zirconia framework was veneered by feldspathic porcelain and occlusion balance was checked. Definitive zirconia_crown bridge prosthesis was fabricated using computer aided design/computer-assisted manufacturing (CAD/CAM) system (Procera, Nobel Biocare). The patient was given home oral health care instructions, including use of dental floss, interproximal brushes, and an oral mouth rinse.

**Figure 6:**
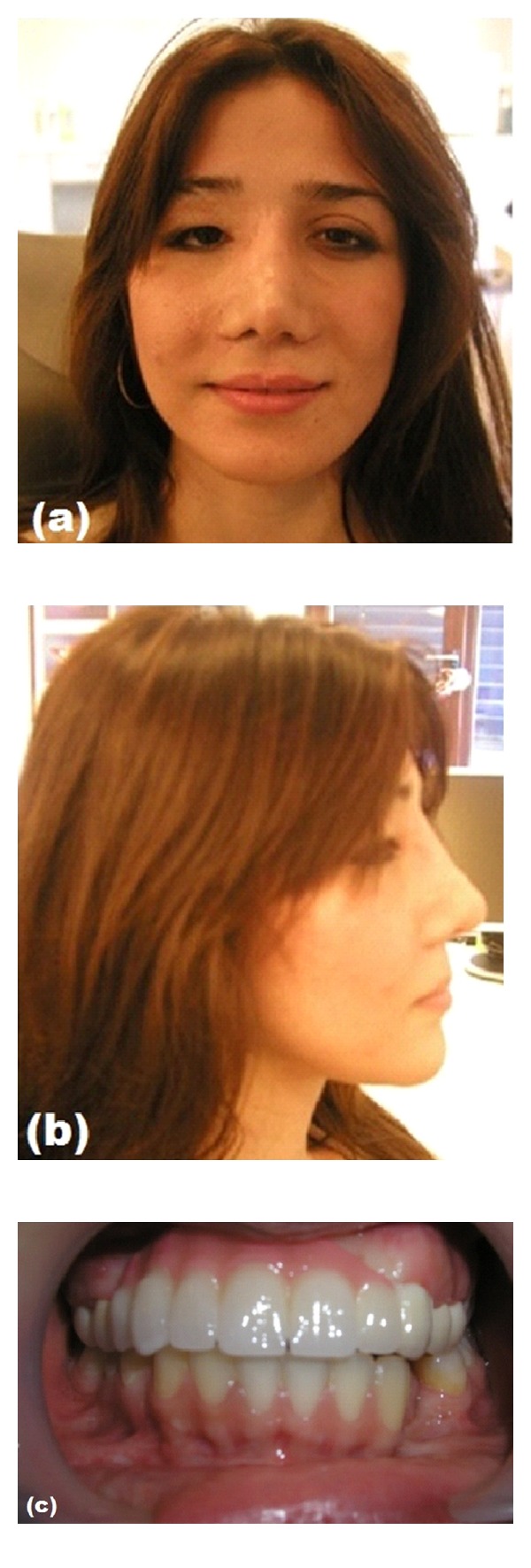
(a, b) Facial view of patient after final treatment with prosthesis, (c) intraoral view of final zirconia-based prosthesis.

The advantages of combined prosthesis included esthetic and biocompatible restoration with zirconia prosthesis. A satisfactory esthetic and functional result was achieved after fixed denture adjustments (Figure 6). After the 1st, 3rd, 6th, and 12th months recall visit, the patient was satisfied with her new appearance and had no functional difficulties during eating, chewing, or swallowing. Speech impairment was eliminated considerably and the patient's profile was improved to a certain degree. In a followup of 5 years period, the prosthesis was stable and there was no evidence for relapse or dysmorphology was found.

## 3. Discussion

Wide maxillofacial defects may create functional and esthetic difficulty as a result of congenital malformations, tumor resections, or trauma [5]. The loss of teeth leads to resorption and remodeling of the alveolar bone and may eventually end with an atrophic residual alveolar ridge [6]. Prosthetic rehabilitation aims to restore anatomic, functional, and esthetic functions when serious soft and hard tissue defects are seen.

Various treatment approaches are often indicated in the planning and treatment of these patients who have severe maxillofacial trauma with acquired maxillary defects [1, 3, 5–8]. These patients usually can be treated to gain normal function and appearance. They are different from patients with congenital maxillary defects only in the abrupt alteration in the physiological processes associated with surgical or traumatic resection of the maxilla [3].

When trauma causes significant defects in the maxillofacial region, fabrication of overdentures is preferred as both hard and soft tissue loss, and lip support can be compensated by means of acrylic resin [9]. However, hard acrylic resin may create a problem through irritation of the fragile mucosa in the mouth after surgical operations. As a treatment procedure, we applied gingival colored porcelain to compensate soft tissue on the anterior maxilla fused to fixed zirconia prosthesis to our patients who had lost their teeth along with bone defect due to facial injury. This kind of modified prosthesis has some advantages such as stability retention and also conforms with the underlying to the hard tissues and supports soft tissues and lip as well.

Patients with such defects experience functional and aesthetic problems which are caused by the edentulous area. Dealing with bone loss in the maxilla and/or mandible, bone grafting of the defect may be necessary in case of implant treatment planning. Extensive soft and hard tissue loss usually requires an implant-supported or retentive prosthesis to obtain adequate facial support and restoration of the oral functions [10]. This treatment option offers an opportunity to enhance the prosthodontic support with advantages such as increased retention, stability, and the preservation of existing hard and soft tissues [11]. Although implant-retained fixed prostheses were desired for this type of large trauma, in this case patient denied the vertical bone augmentation due to repeated surgical procedures which would be needed to provide implant therapy. Therefore, alternative modified combination prosthesis with tissue ceramic and zirconia-based crown prosthesis is applied.

High-strength, full-ceramic system has been recommended with increasing frequent usage for both anterior and posterior restorations. Zirconia has good chemical and physical properties such as high corrosion resistance and low thermal conductivity, high flexural strength (900–1200 MPa), and hardness (1200 Vickers) and also excellent biocompatibility, and optimized esthetics [12]. The adhesion of bacteria on its surface is low [12]. Due to superior flexural strength compared with aluminum oxide, zirconia frameworks for fixed partial dentures for anterior and posterior teeth and for implant-supported restorations are currently being employed. Several *in vitro* reports have demonstrated the superior flexural strength of zirconia, when being compared to other ceramic materials, such as aluminum oxide [12, 13]. In the literature, few long-term clinical studies evaluated systems with zirconium oxide (zirconia) frameworks whose 3 and 4 posterior units have been performed [12, 14]. Papaspyridakos and Lal also have published about an implant supported fixed denture recently [12]. In this case, rehabilitation including 11-unit anterior and posterior tooth supported zirconia fixed prosthesis has been illustrated with 5-year follow-up period; however, in the literature long-term clinical data on longevity of zirconia prostheses are still lacking.

For appropriate treatment procedures of the patients who had wide maxillofacial defects, additional planning, modifications, and treatment considerations are required to evaluate conditions conducive to rehabilitation of both function and esthetics. This includes the establishment of soft tissue support and contour, in addition to tooth and bone health [15]. Among the methods for improving soft tissue deficiencies, tissue compatible porcelain might supply natural mucogingival esthetic appealing and functional lip support on maxillary anterior area. In this case, the desired result in the anterior maxilla as esthetically and functionally and reestablishment of soft tissue support and contour in addition with the teeth and bone health was obtained according to the radiologic and clinical examination resulting in patient's esthetic expectations.

This paper confirms that patients with traumatic injuries have specific treatment needs. Modified prosthetic rehabilitation can enhance the esthetic of the final restoration and provide support for dental rehabilitation, supplying missing teeth, and hard and soft tissue. Through the follow-up period of 6 years, the applied prosthesis was stable and there was no need for additional adjustments nor dysmorphology was observed according to panoramic and periapical radiographs. The patient adapted well to her prosthesis and was satisfied with the final esthetic and functional outcome and reported improvements in both speech and mastication as well.

## 4. Conclusion

In the large defects of the maxilla, detailed presurgical planning and evaluation of each case individually can minimize the difficulty of the prosthetic rehabilitation. It is often necessary for many dental disciplines, including prosthodontics, oral, and maxillofacial surgery and orthodontics to interact in the planning and treatment of patients who have severe maxillofacial trauma. The treatment options should be evaluated according to the patient's need and appropriate case selection with the dental team by careful treatment planning and interdisciplinary cooperation. Prior to finalizing the esthetic design, a treatment plan should include detailed case evaluation and smile analysis as well as patient's expectations.
